# Social, cultural and economic aspects of antimicrobial resistance

**DOI:** 10.2471/BLT.20.275875

**Published:** 2020-12-01

**Authors:** Timo Minssen, Kevin Outterson, Susan Rogers Van Katwyk, Pedro Henrique D Batista, Clare I R Chandler, Francesco Ciabuschi, Stephan Harbarth, Aaron S Kesselheim, Ramanan Laxminarayan, Kathleen Liddell, Michael T Osterholm, Lance Price, Steven J Hoffman

**Affiliations:** a Center for Advanced Studies in Biomedical Innovation Law, University of Copenhagen, Copenhagen, Denmark.; b Social Innovation on Drug Resistance, Boston University, Boston, United States of America (USA).; c Global Strategy Lab, Faculty of Health and Osgoode Law School, York University, 4700 Keele Street, Dahdaleh Building, Suite 2120, Toronto, Ontario, M3J 1P3, Canada.; d Max Planck Institute for Innovation and Competition, Munich, Germany.; e Department of Global Health and Development, London School of Hygiene and Tropical Medicine, London, England.; f Department of Business Studies, Uppsala University, Uppsala, Sweden.; g Antimicrobial Stewardship Program, Geneva University Hospitals, Geneva, Switzerland.; h Program on Regulation, Therapeutics, and Law, Harvard Medical School and Brigham and Women’s Hospital, Boston University, Boston, USA.; i Center for Disease Dynamics, Economics & Policy, Washington, DC, USA.; j Centre for Law, Medicine and Life Sciences, University of Cambridge, Cambridge, England.; k Center for Infectious Disease Research and Policy, University of Minnesota, Minneapolis, USA.; l Milken Institute School of Public Health, George Washington University, Washington, DC, USA.

Although often considered only a medical problem, antimicrobial resistance is an evolutionary challenge accelerated by social, cultural and economic factors that lead to the misuse, overuse and abuse of life-saving antimicrobial medicines. The antimicrobial resistance challenge is compounded by inadequate attention to disease prevention and response, global circulation of people and products, differences in industry and market regulations across countries, and a fragile pipeline of new antibiotics and their alternatives. While the discovery of new antimicrobials will provide temporary solutions, sustainable success requires rigorous social science research that explores the drivers of antimicrobial resistance. These solutions should promote balance between equitable access to, conservation of, and innovation for antimicrobials, adapted to local conditions across the globe.^1^^–^^3^

Effective actions against antimicrobial resistance will need to be informed by insights and evidence from the social sciences, encompassing a broad variety of disciplines. From our perspective, current engagement with the full range of social sciences is inadequate; greater collaboration within and between social science disciplines must be prioritized. Only then can we generate sufficient cross-sectional knowledge to overcome obstacles to addressing antimicrobial resistance, including inequitable access to effective antimicrobials, inadequate sanitation and hygiene infrastructure, disincentives for appropriate use of existing antimicrobials, and insufficient incentive for innovation in developing new antimicrobials.^4^ Collaboration among social scientists from various disciplines is also needed to help anticipate unintended consequences of action, such as inadvertently driving the use of suboptimal antibiotics by raising concerns about resistance.^5^^,^^6^

In the past 15 years, social science research has generated substantial knowledge about the systemic causes of antimicrobial resistance and has identified feasible interventions. After the World Health Assembly adopted the *Global action plan on antimicrobial resistance* in 2015, more than 120 countries developed national action plans. Despite this progress, challenges remain, such as how to scale-up access to antimicrobials without scaling-up resistance, identifying the clinical practices that can reduce antimicrobial use without risking lives, and how to de-link the price of antimicrobials from the cost of their development.^7^^–^^10^ Existing global efforts may be too slow to counter antimicrobial resistance, given the lack of political commitment, the challenge of addressing transboundary collective action problems, and the difficulty in balancing antimicrobial resistance with other global threats.^11^ A range of social science disciplines can provide essential analytic tools for developing solutions for such global challenges.

To encourage collaboration and to address this challenge, we have created the International Network for Antimicrobial Resistance Social Science (INAMRSS).^12^ The network is an open consortium of social science researchers focused on addressing the global challenge of antimicrobial resistance. We believe antimicrobial resistance is only surmountable through efforts that consider social, political and economic factors. We intend to champion social science as part of a broadly defined One Health perspective to inform global initiatives. INAMRSS is a member of the Global AMR R&D Hub stakeholder group, which has built a system to track antimicrobial resistance research investment, but has not yet started monitoring relevant social science research funding. We endorse the recommendation of the United Nations Interagency Coordination Group on Antimicrobial Resistance for creating an Independent Panel on Evidence for Action against such resistance, with appropriate expertise across disciplines including the social sciences, and with a focus on the ways that humans are driving this problem and can contribute to solutions.

As initial steps, INAMRSS strongly recommends several coordinated initiatives to better identify and implement valuable social science insights to support and inform much needed action against antimicrobial resistance. These initiatives include (i) tracking inputs and outputs of social science research, including mapping current research spending, research publications and identifying key gaps; (ii) including social scientists in antimicrobial resistance research teams, panels, and proposals; (iii) exploring social science interventions to address antimicrobial resistance at individual, population and systemic levels; (iv) identifying key requirements for infrastructure support and international coordination, such as the Independent Panel on Evidence for Action Against Antimicrobial Resistance and the Global AMR R&D Hub; and (v) using the data generated above to appropriately fund social science research. Only when we consider the multidisciplinary aspects of the challenge together, will we prevail in addressing antimicrobial resistance.
